# A Randomized Controlled Trial Comparing the Effects of Vilazodone, Escitalopram, and Vortioxetine Monotherapy on the Metabolic Parameters in Patients With Major Depressive Disorder

**DOI:** 10.7759/cureus.67941

**Published:** 2024-08-27

**Authors:** N Simple Santi, Sashi B Biswal, Birendra Narayan Naik, Jyoti Prakash Sahoo, Bhabagrahi Rath

**Affiliations:** 1 Pharmacology, Veer Surendra Sai Institute of Medical Sciences and Research, Burla, IND; 2 Psychiatry, Veer Surendra Sai Institute of Medical Sciences and Research, Burla, IND; 3 Pharmacology, Kalinga Institute of Medical Sciences, Bhubaneswar, IND

**Keywords:** body-mass index, creatinine, liver function, glycemic index, serum lipid profile, metabolism, serotonin dysfunction, blood-glucose, hamilton depression rating scale, age and depression

## Abstract

Background and objectives: The coexistence of major depressive disorder (MDD) and metabolic illness could culminate in an aberrant metabolic profile. Individuals with MDD and type 2 diabetes mellitus (T2DM) are more likely to have impaired metabolic indicators. Effective antidepressant therapy can alleviate depressive symptoms and metabolic abnormalities. We focused on the effects of vilazodone, escitalopram, and vortioxetine on metabolic indices. Our research aimed to examine changes after 16 weeks of intervention in the glycemic indices, serum creatinine, lipid profile, hepatic parameters, and the Hamilton Depression Rating Scale (HDRS) 17-item version.

Methods: A three-arm, randomized, open-label trial with 96 MDD patients was executed. Participants were divided into three distinct groups in a 1:1:1 ratio for 16 weeks and issued tablets of vilazodone (20-40 mg/day), escitalopram (10-20 mg/day), or vortioxetine (5-20 mg/day). Vilazodone and vortioxetine were the two test medications, while escitalopram served as the control. We stratified the participants as non-diabetics and diabetics. Follow-up appointments were slated four weeks after the initial visit. HDRS scores and other metabolic indicators were assessed at each visit in the per-protocol (PP) population. After 12 weeks, glycated hemoglobin (HbA_1c_) levels were measured. Lower HDRS scores indicated that depression-related symptoms had improved. We investigated the relationship between the 16-week differences in the fasting blood sugar (FBS) and HDRS scores. The Kruskal-Wallis test, Bonferroni correction, and Pearson correlation were all used in our analysis. We registered our trial prospectively in the Clinical Trial Registry of India (CTRI) (2022/07/043808).

Results: Of the 134 people we screened, 119 (81.34%) were deemed eligible. The PP population included 96 (88.07%) of those who completed the 16-week study. The population's average age was 46.3 ± 6.2 years. Across all study groups, the median baseline HDRS score was 30.0 (p = 0.964). At 16 weeks, the equivalent scores dropped to 15.0, 14.0, and 13.0 (p = 0.002). The median FBS levels at baseline and 16 weeks were 100.5, 104.0, and 98.0 (p = 0.491) and 91.5, 98.5, and 91.5 (p = 0.561), respectively. The post hoc analysis manifested no statistically significant changes between any parameters. Except for the reductions in glycemic indices in diabetic patients, no other data differed significantly. There was a positive relationship between FBS and HDRS scores.

Conclusion: Irrespective of the diabetic situation, all three drugs substantially lowered HDRS scores. People with diabetes experienced noticeable declines in glycemic indices. Despite this, the patients' other metabolic indicators showed no significant alterations. We urge additional research with a larger sample size to investigate these medications' long-term impact on various metabolic indicators.

## Introduction

Metabolic illnesses and major depressive disorder (MDD) can coexist through various neurohormonal processes [1-4]. The pronounced prevalence of comorbidity may be rationalized through common biological and genetic pathways [1,3]. Inflammation, the gut microbiota, energy metabolism, mitochondrial function, and the hypothalamic-pituitary-adrenal (HPA) axis constitute the biological pathway [3]. In addition, MDD and physical ailments have many antecedents concerning lifestyle factors (exercise, nutrition, and sleep) [1-3]. Insulin has a knack for crossing the blood-brain barrier (BBB). It can improve dopaminergic communications in the mesolimbic areas, synaptic plasticity, and neurogenesis [5]. Fanelli et al. hypothesized that obesity, type 2 diabetes mellitus (T2DM), and insulin resistance (IR) are related to the onset of cognitive decline in several neuropsychiatric illnesses based on data from genetic and clinical investigations [3,6]. Furthermore, by modulating the brain's immunoinflammatory response, IR-related conditions impede brain neuronal connections and blunt cognitive processing speed [1,3,6]. Furthermore, depression is more likely to develop in people with a raised body mass index (BMI) [3].

MDD has a point prevalence of approximately one in 20 persons and a lifetime risk of approximately one in six persons worldwide [7]. Notwithstanding regional and national variations in frequency, MDD continues to rank among the world's biggest drivers of years lost to disability [8]. Since 1980, many antidepressants with various modes of action have come into our arsenal [7,9]. Nonetheless, the prevalence of depression has paradoxically not dropped in proportion. Nowadays, many effective treatments for MDD are available. However, the prevalence of depression has not decreased in tandem. Ormel et al. introduced the term “treatment-prevalence paradox” (TPP) to characterize this premise [9].

Patients struggling with depression are more susceptible to obesity, diabetes, metabolic syndrome, dyslipidemia, hypertension, and heart diseases as a result of their inadequate physical activity levels [10]. Although there are several classes of efficacious antidepressants available, it is unknown which of them is ideal for maintaining biochemical variables. Furthermore, insufficient information exists concerning the duration of these effects and the impact of circulating 5-hydroxytryptamine (5-HT) [7,9,10]. Therefore, we selected vortioxetine, a modulator of serotonin transporters and receptors; vilazodone, a partial agonist of the 5-HT_1A_ receptor and a selective serotonin receptor inhibitor (SSRI); and escitalopram, an SSRI. We recently looked into these medications' safety and efficacy [11]. Additionally, we investigated the association among treatment outcomes, adherence, and quality of life [12]. The premise that antidepressants with varying modes of action could offer an appealing option for MDD sparked our investigation [13]. Our study's interim analysis has documented these medications' safety and efficacy and their effect on quality of life [14-17].

During the 16-week study period, the following parameters were measured and compared: fasting blood sugar (FBS), the Hamilton Depression Rating Scale (HDRS) score [18], serum creatinine, body mass index (BMI), serum total cholesterol, triglyceride, high- and low-density lipoprotein cholesterols (LDL-c and LDL-c), liver enzymes like AST and ALT, serum albumin, and bilirubin. HbA_1c_ variations were examined at 12 weeks. Furthermore, we correlated the 16-week changes in HDRS and FBS levels.

## Materials and methods

We assessed and compared the effects of vortioxetine, vilazodone, and escitalopram monotherapy on various metabolic markers in MDD patients in this three-arm, open-label, active-controlled, randomized study. The research site was Veer Surendra Sai Institute of Medical Sciences and Research (VIMSAR) in Burla, India, from July 2022 to December 2023. We had ethical permission (029-2022/I-S-T/03) from the Institutional Ethics Committee before the trial's commencement. In writing, each participant provided informed consent prior to the enrollment procedure. We registered prospectively in CTRI (2022/07/043808) for our study. The research adhered to institutional standards, good clinical practices, and the Declaration of Helsinki.

Study participants

In this study, we included participants who had been diagnosed with MDD and had an HDRS score of 24 or higher. Any documented allergy to research medications, chronic kidney failure, organic brain diseases, psychotic symptoms, and any thrombo-embolic episode within the last six months were all considered exclusion criteria. Furthermore, nursing or pregnant moms were not included in this study. Participants were empowered to withdraw their consent at any time.

Study design and endpoints

In this trial, vortioxetine and vilazodone were the experimental drugs, whereas escitalopram was the control. To ensure randomization, the qualified participants were divided into three distinct groups (A, B, and C). A 1:1:1 allocation ratio was in place. The participants in groups A, B, and C received tablets of vilazodone (20-40 mg), escitalopram (10-20 mg), and vortioxetine (5-20 mg), respectively. We adopted permuted block randomization using blocks of 12 and 24 sizes. Based on the MDD duration and gender, we stratified the randomization.

At 16 weeks, the following parameters were assessed and compared: serum creatinine, BMI, HDRS score, FBS, serum total cholesterol, triglyceride, HDL-c, LDL-c, serum albumin, bilirubin, AST, and ALT. After 12 weeks, we analyzed the changes in HbA_1c_. We also examined the correlation between the changes in FBS and HDRS values at 16 weeks. The per-protocol (PP) population was the primary subject of the analyses. To facilitate comprehension of the drug effects, we segregated the subjects into those with and without T2DM.

Study procedure

A course of antidepressant tablets, consisting of vilazodone 20-40 mg once daily (group A), escitalopram 10-20 mg once daily (group B), or vortioxetine 5-20 mg once daily (group C), was administered to each participant for 16 weeks. The participants did not pay for any of these drugs. The psychiatrist adjusted the dosages as per the clinical outcomes. Drug-switching was not permitted. Every participant thoroughly assessed their physical and mental well-being at the baseline visit. Four, eight, 12, and 16 weeks following the baseline visit, we arranged the follow-up appointments. HDRS scores and the previously described metabolic parameters were gauged at baseline and every four weeks until week 16. HbA_1c_ was measured, nevertheless, only 12 weeks after the baseline visit. We correlated the 16-week differences between the HDRS and FBS values. Lower HDRS scores evidenced a reduction in clinical symptoms and the efficacy of the intervention.

Statistical analysis

We considered a standard deviation (SD) of 2.0 and a mean change of 10.0 in HDRS over the baseline values to calculate the sample size. A beta error of 0.20, or 80% power, and a two-sided alpha error of 0.05 were mandated for 87 cases. We chose 96 cases as the sample size permitted a 10% dropout rate. After the 12-week visits concluded for the first 48 subjects, we ran an interim analysis.

The Shapiro-Wilk test was deployed to confirm the normality of the data distribution. Either the median with interquartile range (IQR) or the mean with SD were shown for the continuous data. Accordingly, they were assessed using either the Kruskal-Wallis test or the analysis of variance (ANOVA). The frequency and proportion were displayed for the categorical data. Furthermore, Pearson's chi-square (ꭓ^2^) test was used for their assessment. Version 4.4.0 of the R software (Vienna, Austria) was utilized for data computation [19]. A p-value of 0.05 or less was considered statistically significant.

## Results

**Figure 1:**
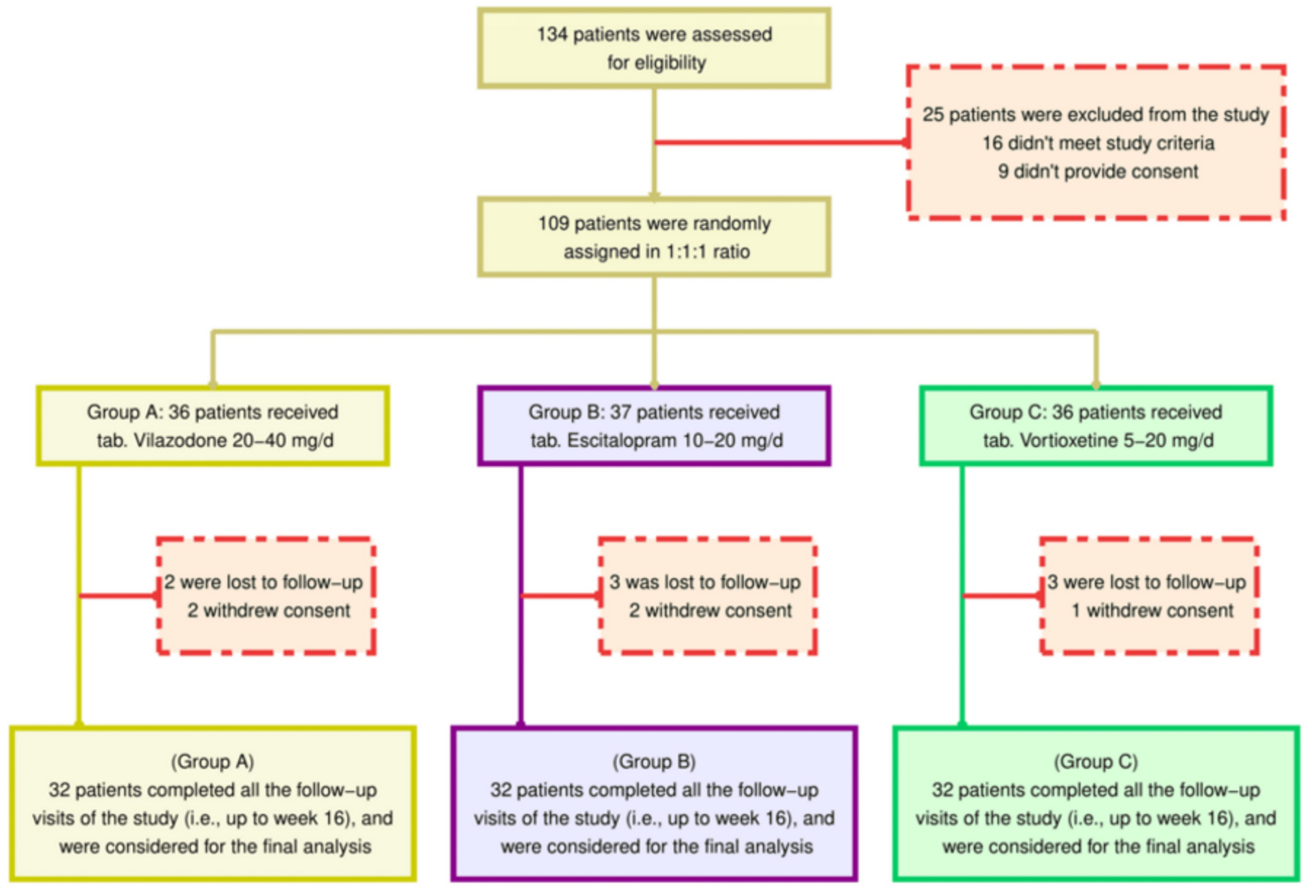
The CONSORT diagram CONSORT: Consolidated standards of reporting trials

One hundred thirty-four patients diagnosed with MDD were screened for this randomized study (Figure 1). Twenty-five subjects were dropped from the study. Sixteen were deemed ineligible, while nine declined the opportunity to participate. A total of 109 patients were assigned at random to the three study groups. Eight did not follow up, and five retracted their consent. The remaining 96 participants were analyzed and found to possess similar baseline traits across the three study groups (Table 1).

**Table 1:** Baseline traits of the study population (n = 96) The median (IQR) or the mean ± SD were selected to depict the continuous variables. n (%) was used to display the category values. BMI: Body mass index; HDRS: Hamilton Depression Rating Scale (17-item version); FBS: Fasting blood sugar; HbA1c: Glycated hemoglobin; LDL-c: low-density lipoprotein cholesterol; HDL-c: high-density lipoprotein cholesterol; AST: aspartate transaminase; ALT: alanine transaminase.

Parameters	Total (n = 96)	Group A Vilazodone (n = 32)	Group B Escitalopram (n = 32)	Group C Vortioxetine (n = 32)	P-value
Age (years)	46.3 ± 6.2	47.1 ± 6.4	46.0 ± 5.5	45.7 ± 6.1	0.143
Age group					
≤ 50 years	64 (66.7%)	23 (71.9%)	20 (62.5%)	21 (65.6%)	0.580
>50 years	32 (33.3%)	9 (28.1%)	12 (37.5%)	11 (34.4%)	
Gender					
Female	48 (50.0%)	16 (50.0%)	16 (50.0%)	16 (50.0%)	1
Male	48 (50.0%)	16 (50.0%)	16 (50.0%)	16 (50.0%)	
Presence of diabetes					
Diabetic	41 (42.7%)	11 (34.4%)	17 (53.1%)	13 (40.6%)	0.634
Non-diabetic	55 (57.3%)	21 (65.6%)	15 (46.9%)	19 (59.4%)	
BMI (kg/m^2^)	27.3 ± 4.8	26.4 ± 4.1	27.7 ± 5.2	27.8 ± 4.5	0.028
HDRS	30.0 (29.0 - 31.0)	30.0 (29.0 - 31.0)	30.0 (29.0-31.0)	30.0 (29.0-31.2)	0.964
FBS (mg/dL)	101.5 (94.0 - 131.5)	100.5 (93.8 - 121.3)	104.0 (95.5 - 130.8)	98.0 (92.8 - 131.0)	0.491
HbA_1c_ (%)	6.5 (5.8 - 7.5)	6.1 (5.8 - 7.6)	6.8 (5.9 - 7.5)	6.6 (5.8 - 7.4)	0.761
Serum creatinine (mg/dL)	0.88 (0.78 - 0.97)	0.90 (0.76 - 0.98)	0.87 (0.82 - 0.96)	0.85 (0.76 - 0.95)	0.509
Serum cholesterol (mg/dL)	134.0 (108.8 - 154.0)	128.0 (109.0 - 144.3)	134.0 (107.8 - 157.5)	144.0 (119.8 - 157.0)	0.243
Serum triglyceride (mg/dL)	126.0 (111.8 - 144.3)	124.0 (104.5 - 142.5)	122.5 (115.8 - 140.5)	129.0 (119.8 - 145.3)	0.440
Serum HDL-c (mg/dL)	37.5 (35.0 - 40.0)	38.0 (35.0 - 40.3)	37.0 (35.0 - 40.3)	37.0 (34.8 - 40.0)	0.594
Serum LDL-c (mg/dL)	141.0 (113.5 - 169.3)	141.0 (120.8 - 162.3)	136.0 (107.0 - 169.8)	148.5 (115.8 - 173.8)	0.489
Serum AST (IU/L)	30.5 (23.8 - 37.0)	33.0 (26.8 - 37.3)	29.0 (22.0-35.8)	30.0 (23.8-35.3)	0.588
Serum ALT (IU/L)	30.0 (23.0 - 37.0)	30.5 (26.0 - 38.0)	31.5 (22.5-37.0)	29.0 (22.0-35.3)	0.453
Serum albumin (g/dL)	4.5 (4.2 - 4.8)	4.6 (4.4 - 4.8)	4.4 (4.2 - 4.8)	4.5 (4.2 - 4.8)	0.881
Serum bilirubin (mg/dL)	0.31 (0.23 - 0.39)	0.33 (0.26 - 0.42)	0.28 (0.23 - 0.34)	0.29 (0.23 - 0.38)	0.232

**Figure 2:**
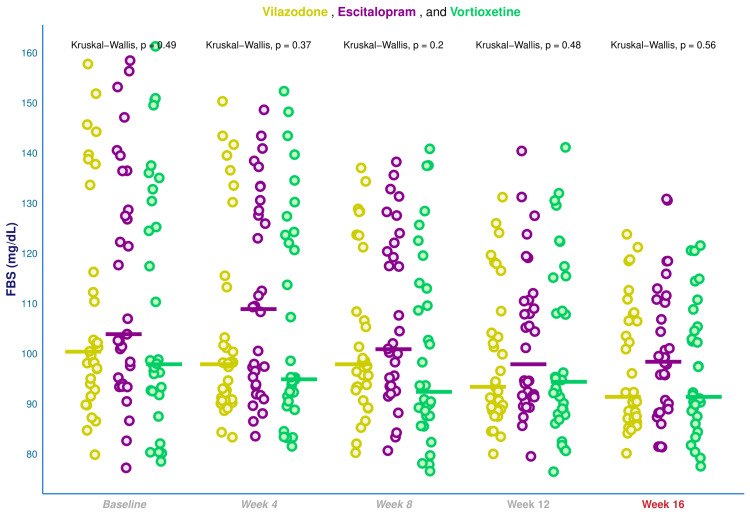
Fasting blood sugar values of the study participants The jitter plots reflect the FBS data of all study subjects at all time points of assessments. The bold horizontal lines denote corresponding medians. The intergroup analyses at every appointment were computed by applying the Kruskal-Wallis test. FBS: fasting blood sugar

Figure 2 displays the FBS values of the study population. The median FBS values at baseline of the three groups’ participants were 100.5 (93.8-121.3), 104.0 (95.5-130.8), and 98.0 (92.8-131.5), respectively (p = 0.491). The corresponding median FBS values at four weeks were 98.0 (91.0-114.5), 109.0 (94.0-129.3), and 95.0 (90.8-124.0), respectively (p = 0.372). After eight weeks, the median FBS values were 98.0 (93.0-111.5), 101.0 (93.8-120.5), and 92.5 (87.0-113.3), respectively (p = 0.200). The median FBS values of the three groups’ participants after 12 weeks of intervention were changed to 93.5 (89.0-105.3), 98.0 (91.8-111.0), and 94.5 (88.0-115.3), respectively (p = 0.479). After 16 weeks, the median FBS values were 91.5 (87.0-107.3), 98.5 (89.0-110.3), and 91.5 (88.8-105.3), respectively (p = 0.561). The trial participants' blood glucose levels steadily decreased, except for a brief upsurge in the median FBS value within the escitalopram group during week 4. When juxtaposed to those in the control group, participants in both test groups experienced clinically significant but not statistically significant declines in their FBS values. We leveraged the Bonferroni test for the post hoc analysis. Supplementary table showcases the subgroup comparison of FBS values in the context of glycemic state at the initial and final visits.

**Figure 3:**
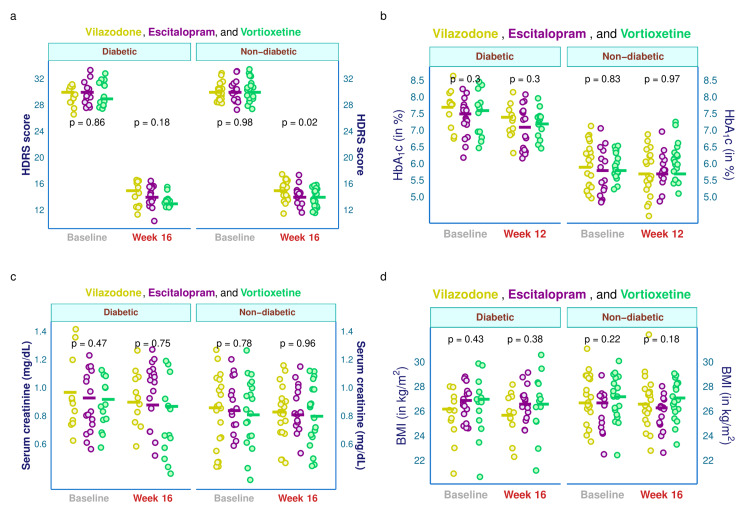
Clinical parameters of diabetic and non-diabetic participants The jitter plots (a, c, d) illustrate HDRS, serum creatinine, and BMI values of diabetic and non-diabetic subjects at baseline and week 16. (b) HbA1c values at baseline and week 12. The intergroup comparisons were performed through the Kruskal-Wallis test. HDRS: Hamilton Depression Rating Scale, 17-item version; HbA1c: glycated hemoglobin; BMI: body mass index.

The HDRS scores, HbA_1c_, serum creatinine, and BMI of the study participants are illustrated in Figure 3. This figure's four plots were faceted according to the baseline visit's glycemic state. Supplemental table provides a comprehensive subgroup assessment of these traits based on the blood glucose levels at the initial and final visit. The median HDRS scores of diabetic individuals in the three study groups were 30.0, 30.0, and 29.0, respectively (p = 0.860). After 16 weeks, these values were reduced to 15.0, 14.0, and 13.0, respectively (p = 0.182). The median baseline HDRS value for the non-diabetic participants was 30.0 irrespective of their study group (p = 0.983), which after 16 weeks got reduced to 15.0, 14.0, and 14.0, respectively (p = 0.020) (Figure 3a). The median baseline HbA_1c_ values of diabetic individuals in the three groups were 7.7, 7.5, and 7.6 %, respectively (p = 0.302). After 12 weeks, the median values for this glycemic index were 7.4, 7.1, and 7.2 %, respectively (p = 0.299). The median baseline HbA_1c_ values of non-diabetic individuals were 5.9, 5.8, and 5.8 %, respectively (p = 0.829). After 12 weeks, their median HbA_1c_ value was 5.7 %, irrespective of their study group (p = 0.968) (Figure 3b). The median baseline serum creatinine values of diabetic individuals in the corresponding groups were 0.97, 0.93, and 0.92 mg/dL, respectively (p = 0.471). After 16 weeks, the values were 0.90, 0.88, and 0.87 mg/dL, respectively (p = 0.749). The non-diabetic participants had a median baseline serum creatinine of 0.86, 0.84, and 0.81 mg/dL, respectively (p = 0.778), which changed to 0.83, 0.81 and 0.80 mg/dL, respectively (p = 0.960) after 16 weeks of intervention (Figure 3c). The baseline BMIs of the people with diabetes were 26.2, 26.9, and 27.0 kg/m^2^, respectively (p = 0.431). After 16 weeks, the values were 25.7, 26.6, and 26.6 kg/m^2^, respectively (p = 0.380). The non-diabetic participants had median baseline BMI of 26.7, 26.7, and 27.2 kg/m^2^, respectively (p = 0.222), which were changed to 26.6, 26.3, and 27.1 kg/m^2^, respectively (p = 0.182) after 16 weeks (Figure 3d).

**Figure 4:**
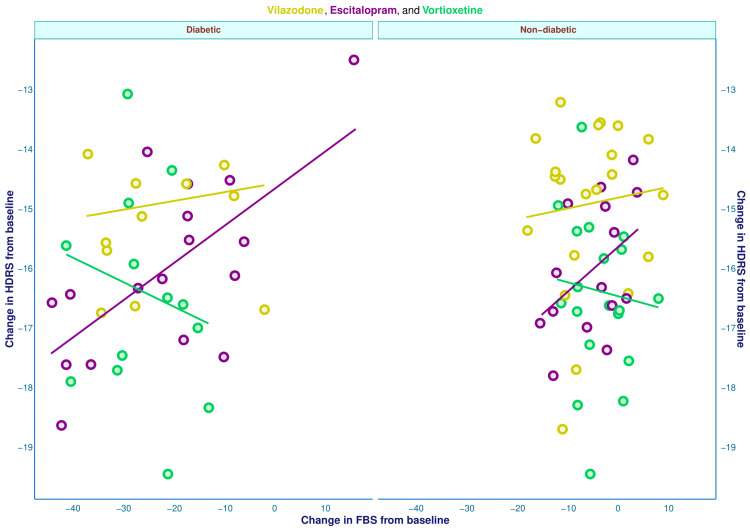
Correlation between differences in HDRS and FBS values of diabetic and non-diabetic participants We used Pearson's correlation to evaluate the associations among the differences in HDRS and FBS values at week 16. The slopes of the straight lines correspond to the correlation coefficients of the respective study groups. DRS: Hamilton Depression Rating Scale (17-item version); FBS: fasting blood sugar.

The correlation between differences in HDRS scores and FBS values at 16 weeks is portrayed in Table 2 and Figure 4. The difference in HDRS score from baseline spanned from -19.0 to -13.0, irrespective of the glycemic status of the participants. The difference in FBS values at 16 weeks among diabetic and non-diabetic participants spanned from -44.0 to 16.0 and -19.0 to 9.0, respectively. There was a positive correlation between the changes in FBS and HDRS scores of the diabetic individuals (r = 0.311, 95% confidence interval (CI): 0.004 to 0.565, p = 0.048). Of the three study groups, group B (those on escitalopram) showed strong positive correlations among diabetic (r = 0.669, 95% CI: 0.279 to 0.870, p = 0.003) and non-diabetic (r = 0.477, 95% CI: -0.047 to 0.795, p = 0.072) subjects. Group A (those on vilazodone) also demonstrated positive correlations among diabetic (r = 0.169, 95% CI: -0.479 to 0.698, p = 0.619) and non-diabetic (r = -0.100, 95% CI: -0.347 to 0.509, p = 0.666) subjects. However, group C (who received vortioxetine) showed negative correlations among diabetic (r = -0.196, 95% CI: -0.674 to 0.398, p = 0.521) and non-diabetic (r = -0.101, 95% CI: -0.531 to 0.369, p = 0.679) individuals.

**Table 2:** Correlation between differences in HDRS and FBS values from baseline We used Pearson's correlation to evaluate the associations among the differences in HDRS and FBS values at week 16. 95% CI: 95% confidence interval; HDRS: Hamilton Depression Rating Scale (17-item version); FBS: fasting blood sugar.

Participants	Correlation coefficient	95% CI	P-value
Total population (n = 96)	0.182	-0.019 to 0.369	0.076
Diabetics (n = 41)	0.311	0.004 to 0.565	0.048
Non-diabetics (n = 55)	0.059	-0.209 to 0.320	0.664
Group A (n = 32)	0.087	-0.269 to 0.423	0.634
Diabetics from group A (n = 11)	0.169	-0.479 to 0.698	0.619
Non-diabetics from group A (n = 21)	0.100	-0.347 to 0.509	0.666
Group B (n = 32)	0.512	0.198 to 0.730	0.003
Diabetics from group B (n = 17)	0.669	0.279 to 0.870	0.003
Non-diabetics from group B (n = 15)	0.477	-0.047 to 0.795	0.072
Group C (n = 32)	-0.079	-0.417 to 0.277	0.665
Diabetics from group C (n = 13)	-0.196	-0.674 to 0.398	0.521
Non-diabetics from group C (n = 19)	-0.101	-0.531 to 0.369	0.679

**Figure 5:**
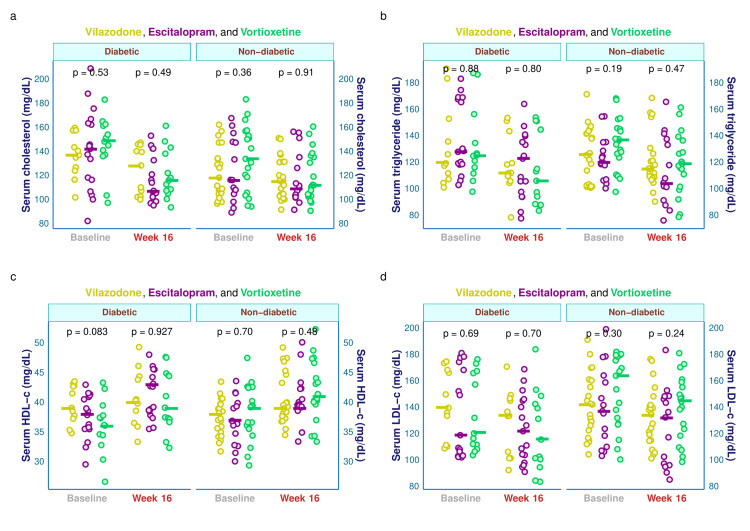
Lipid profiles of diabetic and non-diabetic participants The jitter plots (a-d) display serum total cholesterol, triglyceride, HDL-c, and LDL-c values of diabetic and non-diabetic subjects at baseline and week 16. The intergroup comparisons were done via the Kruskal-Wallis test. LDL-c: low-density lipoprotein cholesterol; HDL-c: high-density lipoprotein cholesterol.

The lipid profiles (serum total cholesterol, triglyceride, HDL-c, and LDL-c) of the study participants are illustrated in Figure 5. This figure's four plots were faceted according to the baseline visit's glycemic state. Supplemental table provides a comprehensive subgroup assessment of these parameters based on the blood glucose levels at the initial and final visit. The median baseline serum cholesterol values of diabetic individuals in the three study groups were 137.0, 142.0, and 149.0 mg/dL, respectively (p = 0.529). After 16 weeks, these values were reduced to 128.0, 107.0, and 116.0 mg/dL, respectively (p = 0.494). The median baseline serum cholesterol for the non-diabetic participants were 118.0, 116.0, and 134.0 mg/dL, respectively (p = 0.361), which after 16 weeks were reduced to 115.0, 109.0, and 112.0 mg/dL, respectively (p = 0.913) (Figure 5a). The median baseline serum triglyceride values of diabetic individuals in the three study groups were 120.0, 128.0, and 125.0 mg/dL, respectively (p = 0.879). After 16 weeks, these values were reduced to 112.0, 123.0, and 106.0 mg/dL, respectively (p = 0.800). The median baseline serum triglyceride for the non-diabetic participants were 126.0, 120.0, and 137.0 mg/dL, respectively (p = 0.193), which after 16 weeks were reduced to 115.0, 104.0, and 119.0 mg/dL, respectively (p = 0.471) (Figure 5b). The median baseline serum HDL-c values of diabetic individuals in the three study groups were 39.0, 38.0, and 36.0 mg/dL, respectively (p = 0.083). After 16 weeks, these values were reduced to 40.0, 43.0, and 39.0 mg/dL, respectively (p = 0.927). The median baseline serum HDL-c for the non-diabetic participants were 38.0, 37.0, and 39.0 mg/dL, respectively (p = 0.700), which after 16 weeks got reduced to 39.0, 39.0, and 41.0 mg/dL, respectively (p = 0.482) (Figure 5c). The median baseline serum LDL-c values of diabetic individuals in the three study groups were 140.0, 119.0, and 121.0 mg/dL, respectively (p = 0.693). After 16 weeks, these values were reduced to 134.0, 122.0, and 116.0 mg/dL, respectively (p = 0.699). The median baseline serum LDL-c for the non-diabetic participants were 142.0, 137.0, and 164.0 mg/dL, respectively (p = 0.304), which after 16 weeks were reduced to 134.0, 132.0, and 145.0 mg/dL, respectively (p = 0.241) (Figure 5d).

**Figure 6:**
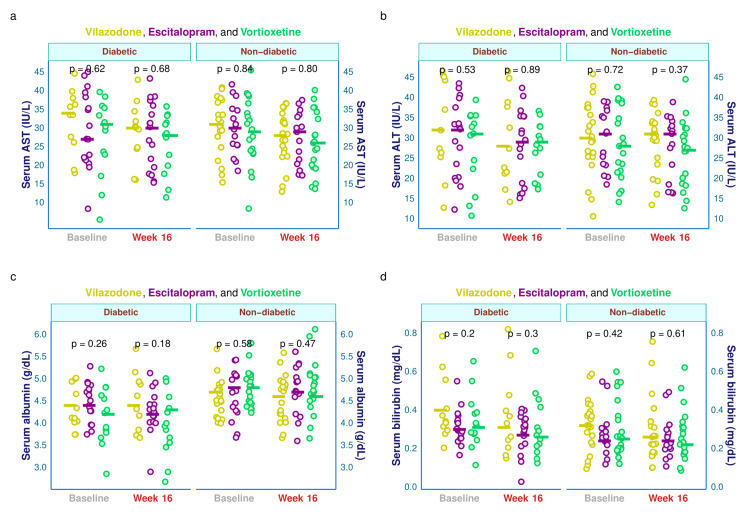
Hepatic parameters of diabetic and non-diabetic participants The jitter plots (a-d) portray serum AST, ALT, albumin, and bilirubin values of diabetic and non-diabetic subjects at baseline and week 16. The intergroup comparisons were performed using the Kruskal-Wallis test. AST: aspartate transaminase; ALT: alanine transaminase.

The hepatic parameters (serum AST, ALT, albumin, and bilirubin) of the study participants are illustrated in Figure 6. This figure's four plots were faceted owing to the baseline visit's glycemic state. Supplemental table displays a comprehensive subgroup assessment of these characteristics based on the blood glucose levels at the initial and final visit. The median baseline serum AST values of diabetic individuals in the three study groups were 34.0, 27.0, and 31.0 IU/L, respectively (p = 0.622). After 16 weeks, these values were changed to 30.0, 30.0, and 28.0 IU/L, respectively (p = 0.680). The median baseline serum AST for the non-diabetic participants were 31.0, 30.0, and 29.0 IU/L, respectively (p = 0.839), which after 16 weeks changed to 28.0, 29.0, and 26.0 IU/L, respectively (p = 0.801) (Figure 6a). The median baseline serum ALT values of diabetic individuals in the three study groups were 32.0, 32.0, and 31.0 IU/L, respectively (p = 0.533). After 16 weeks, these values were changed to 28.0, 29.0, and 29.0 IU/L, respectively (p = 0.889). The median baseline serum ALT for the non-diabetic participants were 30.0, 31.0, and 28.0 IU/L, respectively (p = 0.722), which after 16 weeks changed to 31.0, 31.0, and 27.0 IU/L, respectively (p = 0.371) (Figure 6b). The median baseline serum albumin values of diabetic individuals in the three study groups were 4.4, 4.4, and 4.2 g/dL, respectively (p = 0.258). After 16 weeks, these values were changed to 4.4, 4.2, and 4.3 g/dL, respectively (p = 0.183). The median baseline serum albumin for the non-diabetic participants were 4.7, 4.8, and 4.8 g/dL, respectively (p = 0.580), which after 16 weeks changed to 4.6, 4.7, and 4.6 g/dL, respectively (p = 0.469) (Figure 6c). The median baseline serum bilirubin values of diabetic individuals in the three study groups were 0.40, 0.30, and 0.31 mg/dL, respectively (p = 0.200). After 16 weeks, these values were reduced to 0.31, 0.27, and 0.26 mg/dL, respectively (p = 0.300). The median baseline serum bilirubin for the non-diabetic participants were 0.32, 0.24, and 0.25 mg/dL, respectively (p = 0.421), which after 16 weeks were reduced to 0.26, 0.24, and 0.22 mg/dL, respectively (p = 0.609) (Figure 6d).

## Discussion

In this study, vortioxetine significantly lowered the HDRS scores at 16 weeks when weighed against vilazodone and escitalopram. In both diabetic and non-diabetic subjects, the three study drugs exhibited comparable effects on metabolic indicators. Except for those on escitalopram, there was no significant association between the 16-week changes in FBS and HDRS scores. Our HDRS scores and metabolic parameter results matched the findings of two other studies [13,20] and this study's interim analysis [16].

The daily dosages for the experimental cohorts were 20-40 mg of vilazodone and 5-20 mg of vortioxetine, while the control arm participants received 10-20 mg of escitalopram. In contrast to escitalopram, vilazodone has the perk of operating as a partial agonist for the 5-HT_1A_ receptor. Vortioxetine, on the other hand, limits serotonin transport by interfering with its receptors. Compared to the other two groups, we observed that the vortioxetine group had statistically significant improvements in depressive symptoms and quality of life [11,12]. Among the diabetic subjects, all three drugs demonstrated a larger difference from the baseline for all the parameters.

Nonetheless, these observations did not hold for the non-diabetic subjects, except for HDRS scores. The study's interim analysis supported these findings [16]. The visible impact among diabetic individuals could be attributed solely to their deranged baseline values. Li et al. have advocated that obesity elevates leptin and resistin levels and suppresses adiponectin [21]. Hence, a high BMI might exacerbate the risk of diabetes mellitus and depression. This emphasizes the relevance of maintaining a healthy weight [3,21].

The likelihood of having a T2DM diagnosis is twice as high among individuals with MDD as in the general public [22]. HPA axis dysregulations, insulin signaling changes, and inflammation could be considered attributing factors [3,5,6]. Insulin signaling is essential for neurogenesis, neuroprotection, and synaptic plasticity in the brain [22,23]. Recently, Cai et al. revealed that streptozotocin-induced diabetic rats exhibited a substantial drop in hypothalamic 5-HT concentrations [24]. Serum glucose levels additionally influence the proliferation of 5-HT1A receptors within the hypothalamus [24]. This exemplifies the link between T2DM and MDD. The studies mentioned above support the congruence between the co-occurrence of T2DM and MDD and the responses of diabetic subjects to antidepressants.

Recently, Baldwin et al. discovered that vortioxetine did not cause any clinically significant changes in depression symptoms, weight, BMI, or blood glucose levels in diabetic and non-diabetic subjects [25]. In the interim assessment, we discovered a favorable connection between differences in HDRS and FBS values among diabetes patients [16]. Srisurapanont et al. encountered in a recent network meta-analysis that vortioxetine and escitalopram lower HbA_1c_ levels in diabetic patients with MDD [26]. Sansone et al. discovered that SSRIs improve glycemic indices by enhancing insulin sensitivity and release via a 5HT-dependent mechanism [27]. Our findings were consistent with this research. After 16 weeks of antidepressant medication, all study participants' HDRS scores had dropped significantly. Nonetheless, neither of the drugs elicited any major alterations in the metabolic parameters. According to our previous investigations [11,12], effective antidepressant activity necessitates frequent visits, a high quality of life, and optimal medication adherence.

The main strengths of this study were randomization and subgroup analysis. Periodic follow-up appointments and the HDRS scoring system [18] conferred significant perks. Our study, too, had certain limitations. First, the dropouts could be attributed to the non-blinded design. Second, the drugs used in this trial were provided at no cost. The high cost of the study drugs may limit the study findings' practical utility. Third, depression has multiple features and causes. It is difficult to determine the effectiveness of long-term therapy with antidepressants in real life.

## Conclusions

Regardless of the participant's glycemic state, all three trial medications significantly decreased HDRS scores. It depicted their effectiveness in treating MDD in diabetic and non-diabetic people. The diabetic subjects achieved clinically significant drops in FBS and HbA1c levels. Nonetheless, the subjects' other metabolic parameters showed no significant changes. We recommend more research with a larger sample size to assess these drugs' long-term effects on several metabolic markers.

## Appendices

**Table 3:** Clinical parameters of the diabetic and non-diabetic participants The continuous variables were depicted as the median and IQR. The Kruskal-Wallis test was employed for the intergroup comparison. BMI: Body mass index, HDRS: Hamilton Depression Rating Scale (17-item version); FBS: Fasting blood sugar; HbA1c: glycated hemoglobin; LDL-c: low-density lipoprotein cholesterol; HDL-c: high-density lipoprotein cholesterol; AST: aspartate transaminase; ALT: alanine transaminase.

Parameter	Timepoint of assessment	Presence of diabetes	Study group			P-value
			A (Vilazodone)	B (Escitalopram)	C (Vortioxetine)	
HDRS	Baseline	Diabetic	30.0 (28.5 - 31.0)	30.0 (29.0-31.0)	29.0 (28.0-31.0)	0.860
		Non-diabetic	30.0 (29.0 - 31.0)	30.0 (29.0-31.0)	30.0 (29.0-31.5)	0.983
	16 weeks	Diabetic	15.0 (13.0 - 16.0)	14.0 (13.0 - 15.0)	13.0 (13.0 - 14.0)	0.182
		Non-diabetic	15.0 (15.0 - 16.0)	14.0 (13.5 - 15.0)	14.0 (13.0 - 15.0)	0.020
FBS (mg/dL)	Baseline	Diabetic	139.0 (125.5 - 145.5)	129.0 (121.0 - 141.0)	135.0 (125.0 - 149.0)	0.730
		Non-diabetic	97.0 (90.0 - 100.0)	94.0 (92.0 - 99.5)	94.0 (85.0 - 97.5)	0.341
	16 weeks	Diabetic	111.0 (107.5 - 119.0)	110.0 (101.0 - 116.0)	109.0 (103.0 - 115.0)	0.519
		Non-diabetic	88.0 (86.0 - 91.0)	89.0 (87.0 - 93.5)	89.0 (83.5 - 91.0)	0.882
HbA_1c_ (%)	Baseline	Diabetic	7.70 (7.65 - 7.85)	7.50 (7.10 - 7.80)	7.60 (7.30 - 7.80)	0.302
		Non-diabetic	5.90 (5.70 - 6.10)	5.80 (5.40 - 6.25)	5.80 (5.60 - 6.10)	0.829
	12 weeks	Diabetic	7.40 (7.25 - 7.55)	7.10 (6.90 - 7.40)	7.20 (7.10 - 7.50)	0.299
		Non-diabetic	5.70 (5.50 - 5.90)	5.70 (5.35 - 6.10)	5.70 (5.55 - 6.05)	0.968
Serum creatinine (mg/dL)	Baseline	Diabetic	0.97 (0.87 - 1.04)	0.93 (0.83 - 0.99)	0.92 (0.76 - 0.99)	0.471
		Non-diabetic	0.86 (0.74 - 0.96)	0.84 (0.81 - 0.88)	0.81 (0.75 - 0.93)	0.778
	16 weeks	Diabetic	0.90 (0.83 - 0.98)	0.88 (0.81 - 0.93)	0.87 (0.76 - 0.94)	0.749
		Non-diabetic	0.83 (0.68 - 0.89)	0.81 (0.77 - 0.84)	0.80 (0.76 - 0.86)	0.960
BMI (kg/m^2^)	Baseline	Diabetic	26.2 (25.2 - 26.9)	26.9 (25.7 - 27.6)	27.0 (25.2 - 28.0)	0.431
		Non-diabetic	26.7 (26.2 - 28.2)	26.7 (24.8 - 27.1)	27.2 (26.0 - 28.3)	0.222
	16 weeks	Diabetic	25.7 (25.2 - 26.9)	26.6 (25.9 - 27.4)	26.6 (25.1 - 28.1)	0.380
		Non-diabetic	26.6 (26.2 - 28.0)	26.3 (24.8 - 26.9)	27.1 (26.0 - 28.2)	0.182
Serum cholesterol (mg/dL)	Baseline	Diabetic	137.0 (125.5 - 150.0)	142.0 (116.0 - 164.0)	149.0 (138.0 - 161.0)	0.529
		Non-diabetic	118.0 (106.0 - 134.0)	116.0 (106.5 - 148.0)	134.0 (112.5 - 155.0)	0.361
	16 weeks	Diabetic	128.0 (105.5 - 143.0)	107.0 (102.0 - 133.0)	116.0 (107.0 - 138.0)	0.494
		Non-diabetic	115.0 (104.0 - 131.0)	109.0 (102.5 - 131.0)	112.0 (103.5 - 136.0)	0.913
Serum triglyceride (mg/dL)	Baseline	Diabetic	120.0 (109.5 - 144.5)	128.0 (119.0 - 168.0)	125.0 (116. - 134.0)	0.879
		Non-diabetic	126.0 (103.0 - 142.0)	120.0 (112.5 - 132.0)	137.0 (125.0 - 145.5)	0.193
	16 weeks	Diabetic	112.0 (107.5 - 131.0)	123.0 (97.0 - 136.0)	106.0 (89.0 - 145.0)	0.800
		Non-diabetic	115.0 (109.0 - 127.0)	104.0 (96.5 - 135.5)	119.0 (106.0 - 132.0)	0.471
Serum HDL-c (mg/dL)	Baseline	Diabetic	39.0 (37.5 - 43.0)	38.0 (35.0 - 41.0)	36.0 (34.0 - 38.0)	0.083
		Non-diabetic	38.0 (35.0 - 39.0)	37.0 (34.0 - 39.5)	39.0 (35.5 - 41.0)	0.700
	16 weeks	Diabetic	40.0 (37.5 - 44.5)	43.0 (38.0 - 44.0)	39.0 (37.0 - 45.0)	0.927
		Non-diabetic	39.0 (38.0 - 44.0)	39.0 (39.0 - 42.5)	41.0 (40.0 - 45.5)	0.482
Serum LDL-c (mg/dL)	Baseline	Diabetic	140.0 (122.5 - 167.5)	119.0 (104.0 - 175.0)	121.0 (111.0 - 166.0)	0.693
		Non-diabetic	142.0 (122.0 - 161.0)	137.0 (114.0 - 161.5)	164.0 (130.0 - 178.0)	0.304
	16 weeks	Diabetic	134.0 (104.0 - 146.0)	122.0 (104.0 - 145.0)	116.0 (100.0 - 142.0)	0.699
		Non-diabetic	134.0 (115.0 - 142.0)	132.0 (97.0 - 147.0)	145.0 (120.5 - 157.0)	0.241
Serum AST (IU/L)	Baseline	Diabetic	34.0 (26.5 - 37.0)	27.0 (21.0 - 38.0)	31.0 (23.0 - 35.0)	0.622
		Non-diabetic	31.0 (28.0 - 37.0)	30.0 (25.5 - 35.0)	29.0 (24.0 - 35.5)	0.839
	16 weeks	Diabetic	30.0 (24.5 - 33.5)	30.0 (19.0 - 36.0)	28.0 (20.0 - 32.0)	0.680
		Non-diabetic	28.0 (24.0 - 32.0)	29.0 (21.5 - 33.5)	26.0 (20.0 - 33.5)	0.801
Serum ALT (IU/L)	Baseline	Diabetic	32.0 (26.5 - 44.5)	32.0 (21.0 - 38.0)	31.0 (22.0 - 35.0)	0.533
		Non-diabetic	30.0 (26.0 - 38.0)	31.0 (23.5 - 36.5)	28.0 (22.0 - 36.5)	0.722
	16 weeks	Diabetic	28.0 (21.0 - 38.5)	29.0 (19.0 - 35.0)	29.0 (21.0 - 33.0)	0.889
		Non-diabetic	31.0 (25.0 - 34.0)	31.0 (20.5 - 33.5)	27.0 (19.5 - 31.5)	0.371
Serum albumin (g/dL)	Baseline	Diabetic	4.40 (4.05 - 4.65)	4.40 (4.10 - 4.50)	4.20 (4.00 - 4.30)	0.258
		Non-diabetic	4.70 (4.40 - 4.90)	4.80 (4.35 - 5.00)	4.80 (4.50 - 4.90)	0.580
	16 weeks	Diabetic	4.40 (4.35 - 4.70)	4.20 (4.10 - 4.80)	4.30 (3.60 - 4.50)	0.183
		Non-diabetic	4.60 (4.40 - 4.80)	4.70 (4.50 - 4.95)	4.60 (4.60 - 5.10)	0.469
Serum bilirubin (mg/dL)	Baseline	Diabetic	0.40 (0.29 - 0.51)	0.30 (0.24 - 0.36)	0.31 (0.23 - 0.38)	0.200
		Non-diabetic	0.32 (0.23 - 0.34)	0.24 (0.21 - 0.33)	0.25 (0.24 - 0.38)	0.421
	16 weeks	Diabetic	0.31 (0.28 - 0.44)	0.27 (0.23 - 0.32)	0.26 (0.21 - 0.32)	0.300
		Non-diabetic	0.26 (0.20 - 0.36)	0.24 (0.21 - 0.27)	0.22 (0.19 - 0.34)	0.609
